# Comparison of reference management software with new artificial intelligence-based tools

**DOI:** 10.3352/jeehp.2026.23.2

**Published:** 2026-01-15

**Authors:** Jae Gyeong Jin, Seung Gyu Lee, Jea Hyeun Park, Jang Won Han, Jae Young Kim, Jungirl Seok, Jeong-Ju Yoo

**Affiliations:** 1 Research Factory and Publication Inc., Incheon, Korea; 2 Division of Gastroenterology and Hepatology, Department of Internal Medicine, Soonchunhyang University Bucheon Hospital, Soonchunhyang University School of Medicine, Bucheon, Korea; 3 Department of Otorhinolaryngology-Head and Neck Surgery, Seoul National University Hospital, Seoul National University College of Medicine, Seoul, Korea; The Catholic University of Korea

**Keywords:** Reference management software, AI hallucinations, Academic integrity, CiteWell, PubMed validation

## Abstract

Reference management software (RMS) represents a cornerstone of modern academic writing and publishing. For decades, programs such as EndNote, Zotero, and Mendeley have played central roles in facilitating citation organization, bibliography formatting, and collaborative scholarship. Although each platform has introduced unique innovations, persistent limitations remain, particularly with respect to usability, accessibility, and accuracy. In parallel, the rise of generative artificial intelligence has introduced an unprecedented challenge: the inadvertent inclusion of fabricated or incorrect references mistakenly incorporated into manuscripts. This phenomenon has exposed a critical limitation of traditional RMS platforms, namely their inability to verify reference authenticity. Against this backdrop, new solutions have emerged. One such example is CiteWell (https://citewell.org/), an artificial intelligence (AI)-era RMS that introduces several notable innovations, including PubMed-integrated verification, an intuitive interface for new users, customizable journal-specific styles, and multilingual accessibility. This review provides a comprehensive historical overview of RMS, evaluates the strengths and weaknesses of major platforms, and positions emerging AI-based tools as a new paradigm that combines traditional reference management with essential safeguards for contemporary academic challenges.

## Graphical abstract

[Fig f2-jeehp-23-02]

## Introduction

**Fig. 1.**
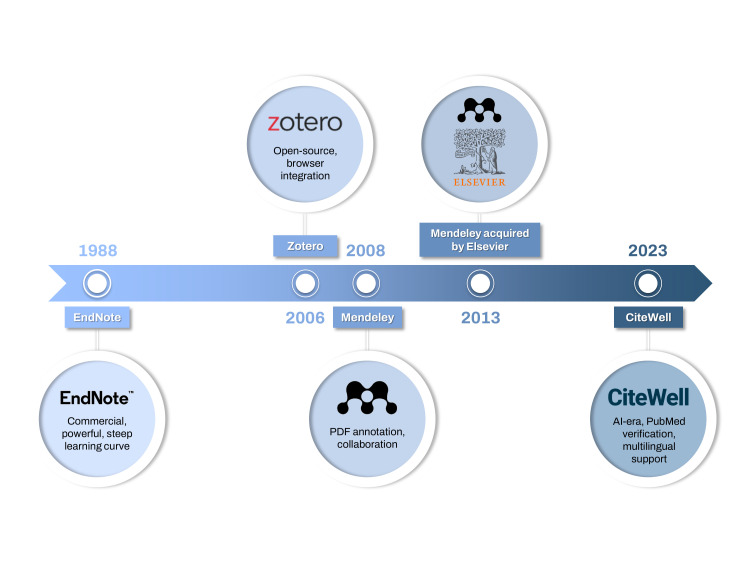
Timeline of reference management software development. A chronological diagram showing milestones: EndNote (1988), Zotero (2006), Mendeley (2008), Elsevier acquisition (2013), and CiteWell (2025).

In modern scholarly research, reference management software (RMS) has evolved from a simple tool for storing citations into an essential component of the entire research workflow [1]. No longer merely a repository, RMS platforms now systematically manage large volumes of academic data and automate the creation of bibliographies across thousands of citation styles, substantially reducing the administrative burden on researchers [2]. EndNote, first released in 1988, has long been a commercial leader in this field and is used by millions of researchers to search bibliographic databases, organize relevant files, and generate reference lists. Accordingly, RMS plays a critical role in helping researchers track the scholarly literature they have consulted while streamlining the writing and revision of scientific manuscripts. The historical development of RMS platforms is illustrated in Fig. 1.

In recent years, large language models (LLMs) have been rapidly integrated into academic research and writing practices. These tools offer substantial efficiency gains by assisting with tasks such as drafting, summarization, and even code generation. However, this convenience has introduced a serious and emerging concern: the generation of “hallucinations,” or fabricated information, which poses a direct threat to academic integrity [3,4]. LLMs do not possess knowledge in the human sense; rather, they function as probabilistic prediction systems trained on massive text corpora to generate the most likely next word, phrase, or sentence [5]. As a result, they may produce fictitious references with plausible author names, article titles, and publication details, mimicking the structure of real citations rather than retrieving verifiable sources. Such errors undermine the reliability of scholarly communication, as authors may unknowingly cite non-existent literature [6].

This review examines conventional reference management software alongside emerging artificial intelligence (AI)-based tools in the context of growing concerns about AI-generated citation errors. We compare widely used platforms with newer AI-assisted solutions, including CiteWell, SemanticCite, and OpenCitations, with particular attention to their approaches to reference validation, transparency, and interoperability.

## A critical analysis of the existing reference management software ecosystem

### EndNote: an enduring powerhouse with structural limitations

EndNote, first released in 1988, is among the oldest and most well-established RMS platforms [7]. For more than 3 decades, it has maintained a strong presence in academic institutions, particularly within the biomedical sciences, where compliance with journal-specific formatting requirements is often stringent [8]. EndNote is widely recognized for its robust library management capabilities [9]. Users can create and maintain large personal databases, organize references into hierarchical groups, and annotate attached documents. Its most influential feature, the “Cite While You Write” plug-in for Microsoft Word, enables seamless insertion of in-text citations and real-time bibliography generation.

EndNote also offers one of the largest collections of citation styles, with more than 7,000 formats that are frequently updated to reflect evolving journal guidelines. This extensive coverage makes the software especially valuable for authors submitting manuscripts to high-impact journals with strict formatting requirements. In addition, EndNote integrates with numerous online databases and supports features such as full-text searching, PDF importing, and automatic metadata extraction.

Despite these strengths, EndNote has several notable drawbacks. The software is commercial and requires a relatively high licensing fee, which can create barriers for individual researchers, students, and institutions in low-resource settings. Its user interface, while powerful, is often perceived as dated and overly complex. For many novice users, effective use of EndNote necessitates formal training through workshops or institutional tutorials. Furthermore, customization of citation styles is cumbersome, as it requires detailed XML-based editing that discourages most users from making modifications. Crucially, EndNote lacks built-in mechanisms for validating the authenticity of references. When incorrect or incomplete metadata are imported, these errors may be propagated throughout manuscripts without warning, potentially resulting in inaccuracies in published work.

### Mendeley: a user-friendly alternative for collaboration

Mendeley was founded in 2008 and quickly distinguished itself by combining reference management with a scholarly social network [10]. After its acquisition by Elsevier in 2013, Mendeley rapidly expanded its user base by offering a hybrid model that blended free RMS functionality with academic networking features [11].

One of Mendeley’s hallmark features is its robust PDF management capability. Users can import, annotate, and organize PDFs directly within the platform [12]. Integrated highlighting, commenting, and tagging tools create an attractive all-in-one environment for reading, organizing, and citing literature. In addition, its recommendation system—based on users’ library contents—facilitates discovery of new research and enhances its appeal for scholars seeking to remain current in their field.

Collaboration was another early strength of Mendeley. Shared folders and group libraries enabled research teams to build collective repositories of articles and references. However, this functionality was partially scaled back in 2021, when Elsevier discontinued several collaborative features, including Mendeley Web Groups.

Although Mendeley continues to provide citation integration with Microsoft Word and LibreOffice, its options for citation style customization are more limited than those offered by Zotero. Moreover, Elsevier’s acquisition raised concerns within the academic community regarding data ownership, long-term sustainability, and potential conflicts of interest. While Mendeley remains widely used—particularly in Europe and Asia—its evolving trajectory has left some researchers uncertain about its future direction.

### Zotero: an open-source standard for flexibility

Zotero emerged in 2006 as an open-source project developed by the Roy Rosenzweig Center for History and New Media [13]. In contrast to EndNote’s commercial model, Zotero provided free access to core reference management functions, effectively democratizing citation software for a global research community. Its emphasis on accessibility and seamless integration into everyday research workflows contributed to widespread adoption, particularly among students and early-career investigators [14].

Zotero is best known for its deep browser integration. With a single click, users can capture bibliographic metadata directly from journal websites, library catalogs, and online repositories. In many cases, Zotero also retrieves the associated PDF and stores it within the user’s library, making it especially attractive to researchers who rely heavily on digital reading and centralized literature management. The platform’s group libraries support collaboration across institutions, while cloud synchronization allows teams to work together in near real time. Although the free tier provides 300 MB of storage, larger libraries can be supported through paid plans.

Another major strength of Zotero lies in its extensibility. A vibrant community of developers contributes plug-ins that expand functionality, including advanced LaTeX integration, enhanced PDF annotation tools, and customized export formats. The ability to modify citation styles through the Citation Style Language framework further affords users a high degree of flexibility.

Nevertheless, Zotero has certain limitations. Metadata imports can be incomplete or inconsistent, particularly when information is scraped from poorly structured webpages, often necessitating manual correction. While integration with Microsoft Word is functional, it is generally less seamless than EndNote’s more mature plug-in. In addition, although Zotero excels in accessibility and flexibility, it lacks some of the organizational depth favored by researchers managing very large and complex libraries.

### Summary of key features and limitations of previous programs

EndNote, Mendeley, and Zotero each exhibit distinct strengths and weaknesses, and the choice of tool often depends on a researcher’s career stage, disciplinary norms, and level of institutional support (Table 1). EndNote offers powerful features and extensive customization options but is comparatively complex and costly. Mendeley emphasizes usability and collaboration but provides limited free cloud storage and has reduced some collaborative functions over time. Zotero, as a free and open-source tool, is highly effective for collecting web-based resources and supporting flexible workflows, though it has certain limitations in PDF management and large-scale library organization. Most importantly, all 3 platforms share a critical shortcoming: the absence of an integrated system for automatically verifying the authenticity of references—a gap that has become particularly consequential in the era of AI-generated content.

## An urgent threat to academic integrity: AI-generated fake citations

### The mechanism of fabrication: from pattern to plausible fiction

LLMs do not “know” whether information is accurate; instead, they generate text based on statistical patterns learned from vast training datasets. This probabilistic prediction mechanism is the root cause of hallucinations, whereby LLMs fabricate references that either do not exist or contain inaccurate bibliographic details [4]. These fake citations often appear legitimate, featuring plausible journal titles, publication years, and formatting conventions, while the authors and article titles themselves are entirely fictitious. Because these references closely mimic real citations, they are difficult to identify through manual review, even by subject-matter experts. This issue is not merely theoretical; for instance, fabricated legal citations generated by an AI chatbot were once included in a professional document submitted in an actual legal case [15].

### Serious and multifaceted consequences

Citing fabricated or incorrect references can lead to severe consequences, including academic penalties, reputational harm, and erosion of trust in the scholarly process [15]. For students, such errors may result in failing an assignment, suspension, or even expulsion. For professionals, they can lead to a loss of credibility among peers and long-lasting damage to their careers. Because research integrity depends fundamentally on accurate citation, papers containing fabricated references may be regarded as unreliable or of diminished scholarly value. Moreover, fabricated references obstruct the scholarly process by complicating source verification, obscuring the origins of information, and impeding the ability to build upon prior work, thereby contributing to the broader spread of misinformation. As a result, affected research outputs may be disregarded or undervalued by the academic community.

### The inadequacy of existing tools

Current market-leading reference management systems were not designed to address the challenge of AI-generated fake citations. These tools were developed primarily to organize, store, and format references, rather than to verify their authenticity. For example, EndNote’s “Find Full Text” feature assists users in retrieving the full text of a known reference but does not confirm whether the underlying citation metadata actually correspond to a real publication. Because AI-generated citations are often difficult to distinguish from legitimate ones, reliance on manual verification becomes both burdensome and time-consuming, particularly for large or complex manuscripts.

## Emerging AI-based approaches to reference integrity

### SemanticCite and citation-context analysis

SemanticCite (https://www.semanticcite.com/) is an AI-based tool designed to enhance understanding of scholarly citations by analyzing their semantic context within scientific texts. Rather than functioning as a traditional reference management system, SemanticCite focuses on how and why a particular source is cited, categorizing citation intent (e.g., background, method, comparison, or critique) using natural language processing techniques. By examining citation contexts at the sentence or paragraph level, SemanticCite provides insights into the rhetorical role of references within a manuscript. However, it does not offer core reference management functionalities such as library organization, citation formatting, or bibliography generation. In addition, SemanticCite does not perform reference authenticity verification against authoritative bibliographic databases.

### Opencitation and open bibliographic infrastructures

OpenCitations (https://opencitations.net/) is an open scholarly infrastructure initiative that provides freely accessible citation data derived from persistent identifiers, primarily digital object identifiers (DOIs). Its central aim is to promote transparency, reproducibility, and openness in scholarly communication by making citation relationships publicly available without commercial restrictions. Rather than serving as an end-user reference management tool, OpenCitations functions as a foundational bibliographic infrastructure that supports downstream applications. Its resources are widely used in meta-research and research evaluation. However, OpenCitations does not provide functionalities typically associated with reference management software, such as personal library organization, citation insertion, or manuscript formatting. Furthermore, although it establishes citation linkages between publications, it does not directly validate the accuracy of reference metadata as entered by authors at the manuscript level.

### CiteWell as an AI-integrated reference management platform

CiteWell (https://citewell.org/) is an AI-integrated reference management platform developed to address the limitations of conventional tools in the context of AI-assisted academic writing. It combines core reference management functions with automated validation mechanisms intended to reduce citation errors introduced by generative AI. A defining feature of CiteWell is its PubMed-integrated reference validation system, which cross-checks citation metadata against an authoritative biomedical database. This process enables identification of non-existent references as well as inconsistencies in bibliographic details, such as publication year or author information. By issuing automated alerts at the reference-entry stage, CiteWell introduces an additional layer of verification that is absent from conventional reference management software.

In terms of interoperability, CiteWell supports widely used bibliographic formats, including RIS and NBIB, allowing users to import and export reference libraries from established platforms such as EndNote, Mendeley, and Zotero. The platform also offers PDF-to-RIS conversion, reducing reliance on manual data entry and facilitating migration from legacy systems. CiteWell provides journal-specific citation formatting and multilingual support, aiming to accommodate diverse publication environments. Compatibility with common writing tools is currently achieved through standard export formats, with direct plug-in–based integration under active development. Despite these strengths, CiteWell has notable limitations. Its validation framework is currently centered on PubMed-indexed literature, which restricts automated verification for non-biomedical sources such as books, conference proceedings, and journals in the humanities. References outside PubMed coverage therefore require manual confirmation. Ongoing development efforts include DOI- and Crossref-based validation to expand disciplinary coverage in future iterations.

## Conclusion

The history of reference management software mirrors the broader trajectory of academic publishing, evolving from labor-intensive manual formatting to commercial standardization, open-source democratization, and, more recently, socially and collaboratively oriented digital platforms. EndNote, Zotero, and Mendeley each represent important milestones in this progression, yet all remain grounded in paradigms that predate the emergence of generative AI.

The rise of AI-assisted writing tools has exposed vulnerabilities that can no longer be overlooked. The proliferation of fabricated references highlights the inadequacy of existing systems in safeguarding the accuracy and reliability of scholarly communication. CiteWell introduces innovations specifically designed to address this challenge, combining intuitive usability with automated verification mechanisms and multilingual accessibility.

By situating CiteWell within the broader continuum of RMS development, its continuity with earlier traditions and its significance as a paradigm shift become clear. For the global research community, CiteWell exemplifies how reference management can evolve to meet the dual imperatives of efficiency and integrity in the AI era.

## Figures and Tables

**Figure f2-jeehp-23-02:**
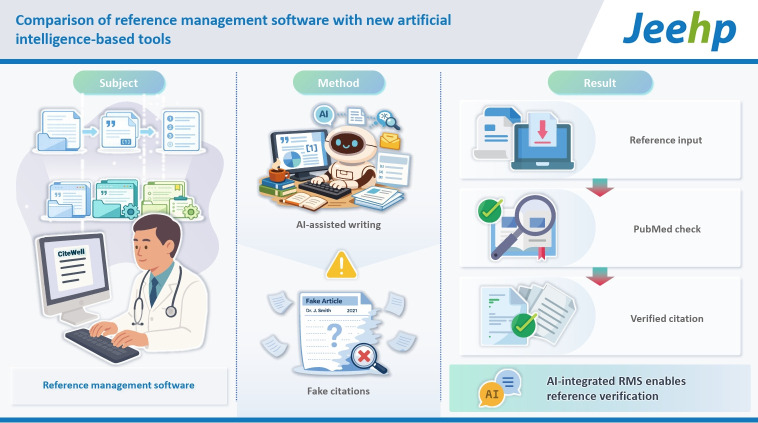


**Table 1.** Comparative features of major reference management softwares

Feature	EndNote	Mendeley	Zotero	SemanticCite	OpenCitations	CiteWell
User interface	Complex, steep learning curve	Intuitive, user-friendly	User-friendly, simple for projects	Web-based analytical dashboard	API-driven, no end-user UI	Highly intuitive, immediately usable
AI citation validation	None	None	None	None	None	PubMed-integrated validation system for authenticity and accuracy
PDF & file conversion	Drag-and-drop PDF import	Drag-and-drop PDF import	PDF import requires manual metadata updates	Not supported	Not supported	PDF-to-RIS or NBIB conversion available
Legacy file support	RIS, XML, and other formats	RIS, BibTeX, EndNote XML	RIS, BibTeX, and others	Not applicable	Not applicable	RIS or NBIB file import and export
Cost & access	Paid, often via institutional license	Free version (2 GB) & paid plans	Free version (300 MB) & paid plans	Free access	Free and open	Very low
Platform	Desktop & iOS app	Desktop, web, mobile apps	Desktop, web, mobile apps	Web-based	Open infrastructure (APIs, datasets)	Web-based
Multilingual support	Primarily English	Primarily English	Primarily English	Primarily English	Language-independent metadata	English, Korean, Japanese, Chinese, and more languages are continuously added
Citation processing speed^a)^	Moderate (1–3 sec)	Moderate (1–3 sec)	Moderate to slow (1–4 sec)	Not applicable	Not applicable	Fast (<1 sec per citation)
Database size limits	Local storage dependent	2 GB free (cloud); paid for more	300 MB free (cloud); paid for more	Not applicable	No predefined limits	Unlimited (cloud-based)
No. of available citation styles	Approximately 500	Approximately 2,000	Approximately 1,000	Not applicable	Not applicable	Approximately 1,000

API, Application Programming Interface; UI, user interface; AI, artificial intelligence.

a) Citation processing speed measured as time required to insert and format a single reference within Word or a web-editor environment.
